# Physiological effects of the combined stresses of freezing-thawing, acid precipitation and deicing salt on alfalfa seedlings

**DOI:** 10.1186/s12870-020-02413-4

**Published:** 2020-05-11

**Authors:** Guozhang Bao, Wenyi Tang, Qirui An, Yaoxin Liu, Jiaqi Tian, Nan Zhao, Saning Zhu

**Affiliations:** Laboratory of Groundwater Resources and Environment of the Ministry of Education, College of New Energy and Environment, Jilin University, Changchun, 130012 China

**Keywords:** Freeze-thaw, Acid precipitation, Deicing salt, Combined stress, Alfalfa

## Abstract

**Background:**

Frequent freeze-thaw phenomena, together with widely used deicing salt and intense acid precipitation, often occur in northeastern China, causing damage to various aspects of plants, such as the permeability of biological membranes, osmotic adjustment, and photosystems. Aiming to explore the resistance of alfalfa to freezing-thawing (F), acid precipitation (A) and deicing salt (D), this study used *Medicago sativa* cv. Dongmu-70 as the experimental material, and the contents of malondialdehyde (MDA), soluble protein, soluble sugars, proline and chlorophyll were evaluated.

**Results:**

As the temperature decreased, the MDA content in the seedlings of the group under combined stress (A-D-F) increased and was significantly higher than that of group F (by 69.48 ~ 136.40%). Compared with those in the control (CK) group, osmotic substances such as soluble sugars and proline in the treatment groups were higher, while the soluble protein content was lower. The chlorophyll contents in the seedlings of the treatment groups were lower than those of the CK group; however, the chlorophyll content displayed a non-significant change during the free-thaw cycle.

**Conclusion:**

Injury to the permeability of the biological membranes and photosystems of alfalfa results from stress. Moreover, alfalfa maintains osmotic balance by adaptively increasing the potential of osmotic substances such as soluble sugars and proline. Furthermore, the influence of stress from freezing-thawing and deicing salt is highly substantial, but the combined stresses of acid precipitation with the two factors mentioned above had little effect on the plants.

## Background

Freezing-thawing refers to the physical geologic phenomenon of the soil layer freezing and melting during late winter and early spring, which occurs in northeastern China [1], and can harm plants [2]. It has been reported that grass-type plants resist low-temperature damage by regulating their proline content [3]. Additionally, considering the frequent and heavy snowfall in winter, deicing salt is widely used because of its low cost, regardless of the severe damage it causes to roadside plants in the form of either runoff or splashing [4, 5]. In addition, the extensive use of coal and oil, as well as the production of sulfur dioxide from coal burning during the winter in northern China, can cause acid precipitation [6]. Moreover, after North America and Europe, China is the third largest area subjected to acid precipitation in the world [7].

Alfalfa is characterized by its adaptability to adversities such as cold, heat, and drought and is widely cultivated in high-latitude areas in China [8], where acid deposition and deicing salt accompanied by freezing-thawing often occur together. A number of previous studies have investigated the resistance of alfalfa under individual factors [9, 10]; however, in the actual environment, plants are often affected by multiple factors. Hence, studying the physiological responses of alfalfa to combined stresses is urgently needed. In this experiment, *Medicago sativa* cv. Dongmu-70 was used as the experimental material to study the physiological responses to combined stresses. Malondialdehyde (MDA) is an end-product of membrane lipid peroxidation, and the MDA content can indicate the extent of cell membrane damage [11]. Soluble protein, soluble sugars and proline are osmotic adjustment substances that can promote cellular bound water content [12]. In addition, chlorophyll content can reflect the photosynthetic intensity and the rate of material synthesis, indicating the level of damage [13]. The above indexes were measured to explore the resistance of alfalfa to acid precipitation, deicing salt and freeze-thaw cycles to provide a theoretical basis for improving the cultivation technology to alleviate damage to plants.

## Results

### Changes in MDA content

**Fig. 1:**
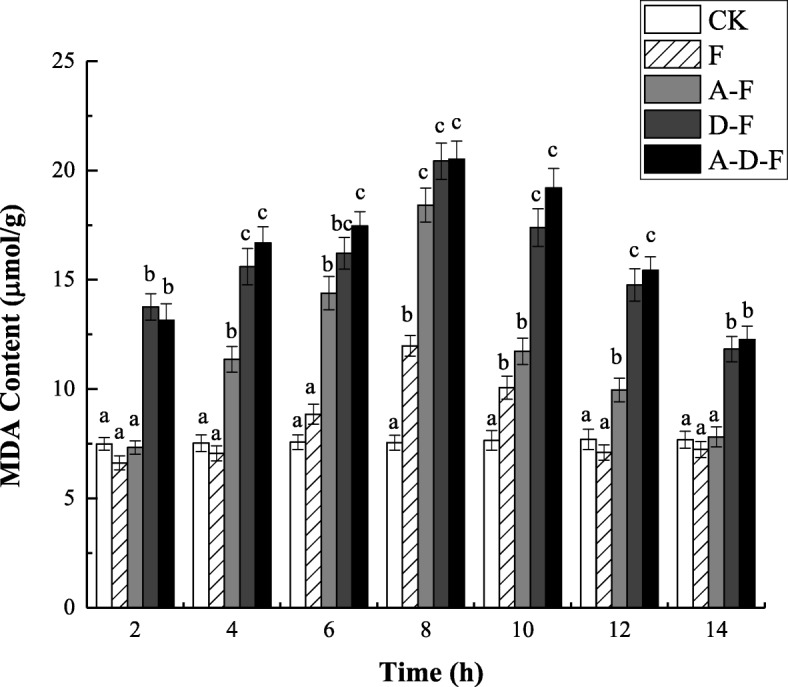
Combined effects of freezing-thawing, deicing salt and acid precipitation on the MDA content in alfalfa seedlings (means ± SEs, *n* = 3). The numbers from 2 ~ 14 h represent different temperatures corresponding to 10, 5, 0, − 5, 0, 5, and 10 °C. CK represents the control group. The letters A, D and F represent the acid precipitation treatment, deicing salt treatment and freeze-thaw treatment, respectively. The different letters indicate significant differences among the various treatments (*P* < 0.05)

Figure 1 shows that the MDA content in the seedlings of groups under combined stresses (A-F, D-F and A-D-F) was higher than that of the group under freeze-thaw (F) stress alone by 7.87 ~ 62.60%, 63.40 ~ 120.96% and 69.48 ~ 136.40%, respectively (see Additional file 1). This indicates that combined stresses cause more intense stress conditions, resulting in the accumulation of MDA in the alfalfa plants. During the thawing period (8 ~ 14 h), the MDA content measured in the seedlings of groups under either combined stresses or freeze-thaw stress alone decreased. When the temperature rose to 10 °C (14 h), the MDA content in the seedlings of groups A-F, D-F and A-D-F decreased by 57.58, 42.10 and 40.20%, respectively (see Additional file 1). It can also be observed from Fig. 1 that under freeze-thaw stress, the MDA content in the seedlings of group A-D-F was significantly higher than that of group A-F (*P* < 0.05), while it showed no significant difference compared with that of group D-F (*P* > 0.05). The above results indicated that the combined stresses had a more significant effect on the MDA content in seedlings than did the freeze-thaw stress alone, and deicing salt stress had a greater impact on the MDA content than did the acid precipitation and freeze-thaw stress.

### Changes in soluble protein content

**Fig. 2:**
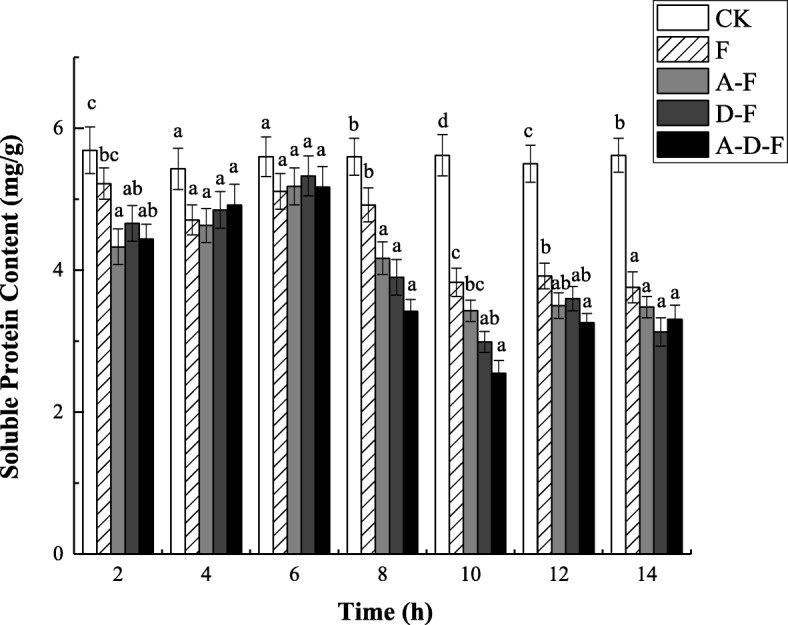
Combined effects of freezing-thawing, deicing salt and acid precipitation on the soluble protein content in alfalfa seedlings (means ± SEs, *n* = 3). The numbers from 2 ~ 14 h represent different temperatures corresponding to 10, 5, 0, − 5, 0, 5, and 10 °C. CK represents the control group. The letters A, D and F represent the acid precipitation treatment, deicing salt treatment and freeze-thaw treatment, respectively. The different letters indicate significant differences among the various treatments (*P* < 0.05)

According to Fig. 2, the soluble protein content in the seedlings of the combined stress groups tended to increase initially but then decrease throughout the whole freeze-thaw cycle, while that of the freeze-thaw stress alone group showed a dynamic decrease. When the temperature decreased to 0 °C (6 h), the soluble protein content in the seedlings of groups under the combined stresses peaked. At the thawing stage (8 ~ 14 h), the soluble protein content in the seedlings of groups under freeze-thaw stress was significantly lower than that of the control (CK) group (*P* < 0.05) (see Additional file 2), which may be attributed to the addition of freeze-thaw stress. However, the content of soluble protein in the seedlings of groups under compound stresses showed no significant difference compared with that of the group under freeze-thaw stress alone, indicating that either acid precipitation stress or deicing salt stress had a lower impact on the soluble protein content. Moreover, during this period, a higher soluble protein content was detected in the freeze-thaw group than in the combined stress groups, indicating that the combined stresses caused more damage to the plants than did the individual stresses.

### Changes in soluble sugar content

**Fig. 3:**
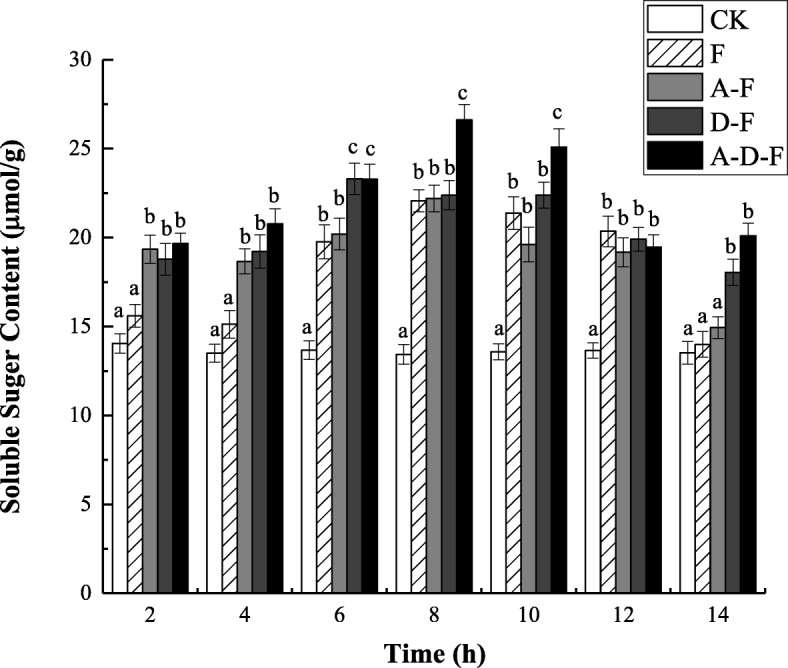
Combined effects of freezing-thawing, deicing salt and acid precipitation on the soluble sugar content in alfalfa seedlings (means ± SEs, *n* = 3). The numbers from 2 ~ 14 h represent different temperatures corresponding to 10, 5, 0, − 5, 0, 5, and 10 °C. CK represents the control group. The letters A, D and F represent the acid precipitation treatment, deicing salt treatment and freeze-thaw treatment, respectively. The different letters indicate significant differences among the various treatments (*P* < 0.05)

Figure 3 shows that the soluble sugar content of each test group was significantly higher than that of the CK group during the freeze-thaw cycle. When the temperature decreased, the soluble sugar content in the seedlings of all groups except the CK group increased and peaked at − 5 °C (8 h). These results demonstrated that in the low-temperature environment, the soluble sugar content in the plants increased significantly, and the plants protected themselves by accumulating a large amount of soluble sugars. The highest soluble sugar content was measured in plants subjected to the combined stresses of freezing-thawing, deicing salt and acid precipitation. During the thawing period (8 ~ 14 h), the soluble sugar content in the seedlings of all the groups except the CK tended to decrease as the temperature increased. Notably, when the temperature rose from − 5 °C (8 h) to 0 °C (10 h), the soluble sugar content in the seedlings of group F was significantly lower than that of group A-D-F (by 17.13% (8 h) and 14.79% (10 h)) (see Additional file 3) (*P* < 0.05), but the soluble sugar content in the seedlings of groups A-F and D-F did not differ significantly from that of group F (*P* > 0.05). These findings indicated that the conditions resulting from the combination of the three stress factors caused the maximum accumulation of soluble sugars in the plants.

### Changes in proline content

**Fig. 4:**
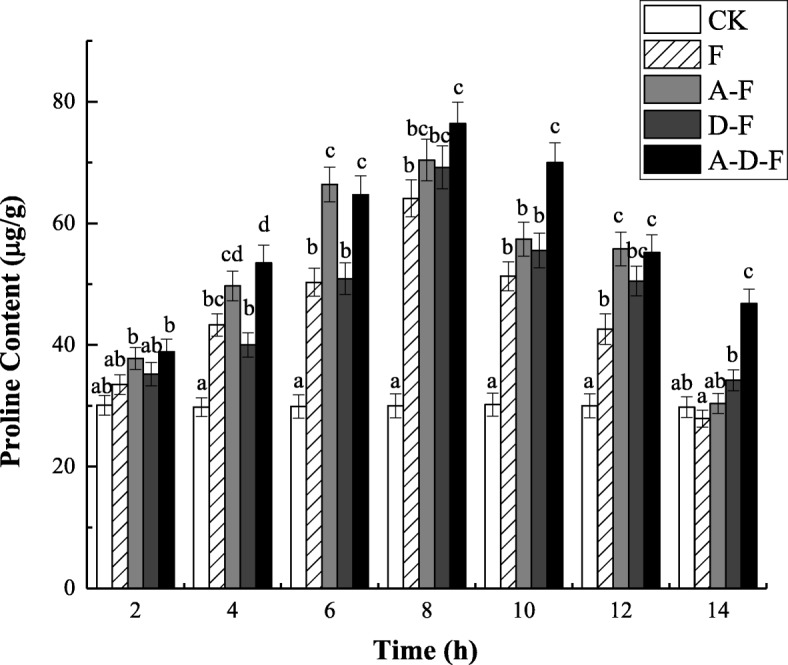
Combined effects of freezing-thawing, deicing salt and acid precipitation on the proline content in alfalfa seedlings (means ± SEs, *n* = 3). The numbers from 2 ~ 14 h represent different temperatures corresponding to 10, 5, 0, − 5, 0, 5, and 10 °C. CK represents the control group. The letters A, D and F represent the acid precipitation treatment, deicing salt treatment and freeze-thaw treatment, respectively. The different letters indicate significant differences among the various treatments (*P* < 0.05)

As shown in Fig. 4, the proline content in the seedlings of the test groups was higher than that of the CK group throughout the whole freeze-thaw period, indicating that stresses resulting from acid precipitation and deicing salt caused an increase in proline content in the plants. During the freezing period, the proline content in the seedlings of groups F, A-F, D-F and A-D-F increased and peaked at − 5 °C (8 h); the contents were 91.34, 86.24, 96.59 and 96.40% higher than those measured at 10 °C (2 h), respectively (see Additional file 4). During the thawing period (8 ~ 14 h), the proline content of groups F, A-F, D-F and A-D-F at − 5 °C (8 h) decreased by 19.97, 18.46, 19.80 and 8.38%, respectively, compared with those measured at 0 °C (10 h) (see Additional file 4). Figure 4 also showed that the proline content was significantly higher in group A-D-F than in the group subjected to only freezing-thawing (*P* < 0.05). In addition, except for that in the CK group, the proline content in the seedlings of the groups under acid precipitation stress was significantly higher than that of the groups not under acid rain stress (*P* < 0.05), which indicated that freeze-thaw stress accompanied by acid precipitation stress resulted in more proline produced in plants to protect themselves.

### Changes in chlorophyll content

**Fig. 5:**
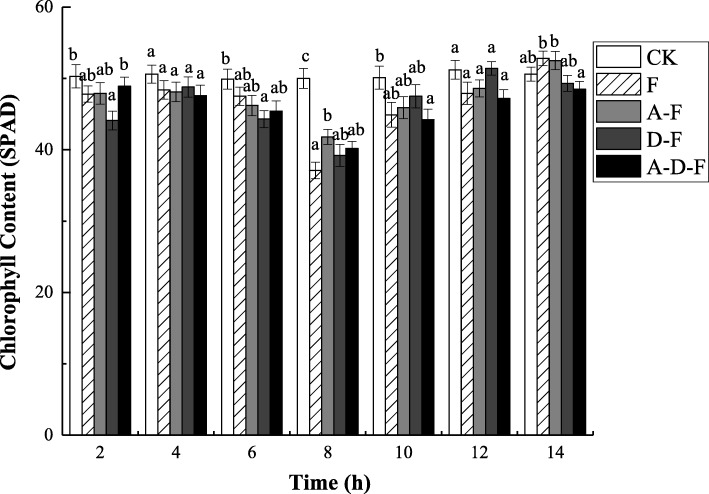
Combined effects of freezing-thawing, deicing salt and acid precipitation on the chlorophyll content in alfalfa seedlings (means ± SEs, *n* = 3). The numbers from 2 ~ 14 h represent different temperatures corresponding to 10, 5, 0, − 5, 0, 5, and 10 °C. CK represents the control group. The letters A, D and F represent the acid precipitation treatment, deicing salt treatment and freeze-thaw treatment, respectively. The different letters indicate significant differences among the various treatments (*P* < 0.05)

During the freeze-thaw period, the chlorophyll content in the seedlings of each experimental group exhibited an initial decrease followed by an increase (Fig. 5). At the freezing stage, the chlorophyll content in groups F, A-F, D-F and A-D-F tended to decrease and reached the minimum value at − 5 °C (8 h), which were 22.38, 12.73, 11.11 and 17.79% lower than those measured at 10 °C (2 h), respectively (see Additional file 5). During the thawing period (8 ~ 14 h), compared with the chlorophyll content measured at − 5 °C (8 h), the content measured at 10 °C (14 h) in the seedlings of groups F, A-F, D-F and A-D-F significantly increased by 42.32, 25.60, 25.77 and 20.65%, respectively (*P* < 0.05) (see Additional file 5). However, there was no significant difference in chlorophyll content among the experimental groups throughout the whole freeze-thaw period (*P* > 0.05).

### Correlation analysis between indexes

Table 1 shows that under freeze-thaw conditions, MDA and proline were significantly positively correlated (*P* < 0.01), each of which was positively correlated with soluble sugars (*P* < 0.05). Chlorophyll was negatively correlated with MDA, proline and soluble sugars. However, there was no significant correlation between protein and the other indicators. The correlations between the indexes of the freeze-thaw + acid precipitation + deicing salt group were similar to those of the freeze-thaw group, but all correlations were highly significant in the former group (*P* < 0.01). These findings indicated that both proline and the soluble sugar content increased with the accumulation of MDA in plants under external stress, while the chlorophyll content decreased.

**Table 1:** Pearson correlation analysis under freeze-thaw (F) and combined (A-D-F) stresses

		MDA	soluble protein	chlorophyll	proline	soluble sugars
Freeze-Thaw Group	MDA	1.000				
	soluble protein	0.090	1.000			
	chlorophyll	−0.867^b^	−0.348	1.000		
	proline	0.887^a^	0.270	−0.904^a^	1.000	
	soluble sugars	0.781^b^	0.016	−0.761^b^	0.855^b^	1.000
Freeze-Thaw +Acid Precipitation +deicing salt Group	MDA	1.000				
	soluble protein	−0.153	1.000			
	chlorophyll	−0.904^a^	0.296	1.000		
	proline	0.946^a^	−0.291	−0.936^a^	1.000	
	soluble sugars	0.895^a^	−0.261	−0.947^a^	0.921^a^	1.000

^a^indicates a significant correlation at the 0.01 level

^b^indicates a significant correlation at the 0.05 level

## Discussion

### Effects of combined stresses on the membrane system

MDA is one of the main products of membrane peroxidation in plants under adverse conditions and can strongly react with various components in the cell, causing cross-linking polymerization of macromolecules such as proteins and nucleic acids [14], leading to membrane structure damage and impaired physiological function [15, 16]. It has been pointed out that the degree of membrane damage caused by the complex stress environment caused by freezing-thawing, acid precipitation and deicing salt could be characterized by changes in MDA content [17]. In this experiment, the damage to the cell membrane caused by three stress factors resulted in the most obvious increase in MDA content at − 5 °C, indicating that the combined stresses caused more severe membrane lipid peroxidation injury to the seedlings than did the individual stresses, which is consistent with published results about the effects of low-temperature stress on plant physiological indexes [18, 19]. Similar to the results of research on *M. sativa* L. under salt stress [20], in this experiment, it was pointed out that salt stress aggravated the damage to the membrane and led to an increase in MDA content.

### Effects of combined stresses on osmotic adjustment substances

Protein is the material basis of life, the most abundant organic macromolecule in plant cells, the basic unit of organic matter that constitutes cells, and the main component of enzymes, which participate in chemical reactions in cells [21]. It can be seen that the soluble protein contents in the seedlings of groups under stress were lower than those in the CK group under freeze-thaw stress, which could be explained by the reduction in the activity of either protein synthetases or other enzymes in plants under adverse conditions, resulting in a decrease in soluble protein content. This result is similar to that presented by Mao and Xu, who reported that salt treatment leads to a decrease in protein content in plant seedlings [11]. The soluble protein content in seedlings increased with decreasing temperature in this experiment, which was attributed to the enhanced expression of related genes, which could effectively stimulate the accumulation of protective substances, reduce cell metabolism and facilitate resistance to low temperature [22]. The reason why the soluble protein decreased during the period in which the temperature decreased to − 5 °C and increased to 10 °C might be that the protein was consumed when the plants adapted to adverse conditions and maintained growth, similar to the results of Fleck et al., who investigated the protein content in winter wheat leaves and their freezing resistance [23].

Free proline is an important osmotic regulator in plants. It has been reported that under stress conditions, the proline mass ratio in plant cells greatly increases, which reduces the cell osmotic potential and helps cells absorb water, thus preventing protoplasm and protein molecules from dehydrating [24]. Hence, a change in the proline content can be used as an indicator of the cold resistance of plants [3]. The results of this experiment showed that the proline content of all groups significantly increased in the low-temperature environment. This is consistent with the research results of Hare et al., who analysed the cumulative effects of osmotic pressure under stress conditions and suggested that proline improved the stress resistance to low temperature [25]. Li et al. not only found that the accumulation of osmotic adjustment substances such as proline can decrease the cellular water potential and maintain osmotic balance but also demonstrated that, to some extent, the more anticold capability plants exhibit, the more significant the change in proline content [26]. Acid precipitation can cause acute injury to leaves and can restrict growth. As such, in this experiment, the proline content was more strongly influenced in the A-D-F group than in the other groups.

Soluble sugars play an important role in the growth cycle of plants. It has been pointed out that under salt stress, plants can accumulate a large number of osmotic regulators, such as soluble sugars and other inorganic salt ions [27], which can reduce the osmotic potential in cells, enhance water absorption and water holding capacity, and maintain cell growth, thus enhancing the ability to resist stress [28, 29]. As a result, an upward trend of soluble sugar content in seedlings was detected in this study, indicating that plants can protect themselves under adverse conditions by accumulating soluble sugars [30]. This may be due to the increasing cell fluid concentration in plants subjected to low-temperature stress, lowering the freezing point and reducing the excessive dehydration of cells; thus, the protoplasm colloid was protected from cold-induced coagulation, enhancing adaptability to low-temperature stress [31]. It was also found that the soluble sugar content of the A-D-F group was much higher than that of the control group, owing to more soluble sugars activating the osmotic pressure regulatory mechanism in plants to resist the combination of stresses [32].

### Effects of combined stresses on chlorophyll content

The pigment in the leaves is designed to absorb light energy and provide energy for plant growth; thus, the responses of chlorophyll content to adverse environments can indicate the ability of photosynthesis [25, 33]. Consistent with the results of this experiment by which the chlorophyll content in the seedlings of group F was reduced, Xin and Browse pointed out that low temperature was one of the main factors that affects photosynthesis of vegetative tissue [34]. However, the effects of other stress factors on chlorophyll and other indicators are also different. The study results of Zhou indicated that photosynthetic indicators such as chlorophyll a, chlorophyll b, and carotenoid contents of *Populus euphratica* leaves under NaCl stress remained relatively stable [35], similar to the present results, in which there was no significant difference among groups A-F, D-F and A-D-F.

## Conclusions

In this paper, alfalfa seedlings were used as experimental material, and the contents of malondialdehyde, soluble protein, soluble sugars, proline, and chlorophyll in the plants were measured to determine the responses of alfalfa to artificial acid precipitation, deicing salt and freezing-thawing. The accumulation of MDA content in alfalfa indicated that the cell membrane system was damaged. Moreover, the increasing content of soluble sugars and proline maintains osmotic balance, which could help plants to resist damage to adverse conditions. However, there was no regular change in chlorophyll content in plants under the combination of stress factors. In summary, the influence of stress from freezing-thawing and deicing salt is highly substantial, while the combined stresses of acid precipitation with the two factors mentioned above has little effect on plants.

## Methods

### Plant materials

Approximately 1000 seeds of Dongmu-70 provided by the Life Sciences of Northeast Normal University of China were selected, soaked in 0.1% acidic KMnO_4_ solution for 2 h, and then rinsed with distilled water. The seeds were then arranged onto trays and covered with two layers of filter paper, and 100 ml of Hoagland nutrient solution was added. After the seeds germinated under dark conditions at 20 °C for 24 h in an MGC-450BP light incubator (Shanghai Yiheng Scientific Instruments Co., Ltd), full and similarly sized seedings were selected, placed on trays of 26 × 18 cm (length × width; 23 lines × 40 seeds per line) and then transferred to an MGC-450BP light incubator (Shanghai Yiheng Scientific Instruments Co., Ltd) under 12-h light (16,500 lx; 25 °C)/12-h dark (0 lx; 15 °C) conditions for 1 week. Eight millilitres of nutrient solution was given every day – 40 ml each morning and evening.

### Stress applications

A deicing salt solution (0.1 M, 1000 ml) and an acid precipitation solution (pH = 4.5 (sulfuric acid:nitric acid = 3:1), 1000 ml) were prepared. The seedlings were then evenly divided into eight groups: as A-D-F, A-F, D-F, A-D, A, D, F and CK (Table 2). Seven millilitres of acid precipitation solution and 18 ml of deicing salt solution were added to groups A-D-F and A-D, respectively, 7 ml of acid precipitation solution and 18 ml of distilled water were added to groups A and A-F, respectively, and 18 ml of deicing salt and 7 ml of distilled water were added to groups D and D-F, respectively. Twenty-five millilitres of distilled water was added to the F and CK groups. After the reagents were added, all the test group plants were placed in a light incubator under 12-h light (16,500 lx; 25 °C)/12-h dark (0 lx; 15 °C) conditions for 2 d and then were removed for the freeze-thaw treatment.

**Table 2:** Experimental design of groups under acid precipitation (a), deicing salt (D) and freeze-thaw (F) stress

	A-D-F	A-F	D-F	F	A-D	A	D	CK
Acid precipitation	+	+	–	–	+	+	–	–
Deicing salt	+	–	+	–	+	–	+	–
Freeze-thaw	+	+	+	+	–	–	–	–

+ add stress, − no stress

The A-D-F, D-F, A-F and F groups were subjected to freeze-thaw treatment, and the seedlings were put into a BPHJ-120A high-low temperature test chamber (Shanghai Yiheng Scientific Instruments Co., Ltd) to carry out a freeze-thaw cycle for a period of 14 h, with a constant temperature curve including 10, 5, 0, − 5, 0, 5 and 10 °C (2 ~ 14 h). The initial temperature of the BPHJ-120A high-low temperature growth chamber (Shanghai Yiheng Scientific Instruments Co., Ltd) was 15 °C, which was close to room temperature at night. The temperature dropped steadily to − 5 °C at a rate of 0.5 °C every 12 min (approximately 0.04 °C/min), and then the temperature rose from − 5 to 10 °C at a rate of 0.5 °C every 12 min (approximately 0.04 °C/min). Groups A-D, D, A, and CK were maintained in an MGC-450BP light incubator (Shanghai Yiheng Scientific Instruments Co., Ltd) with steady light conditions. At each temperature, 9-d-old samples were taken from each treatment group at random according to the amount required for the measurements, i.e., 0.5 g for measuring the malondialdehyde (MDA) and soluble sugar content, 0.1 g for the soluble protein content, and 0.5 g for the proline content.

### Biochemical characterization

The MDA and soluble sugar contents were measured with the thiobarbituric acid (TBA) chromatometry method [36]. Leaf samples (0.5 g) were ground into a homogenate with 5 ml 10% trichloroacetic acid (TCA) solution. After centrifugation (TDL-40B, Anting Scientific Instrument Factory, Shanghai) at 4000 rpm for 10 min, 2 ml of the supernatant was transferred to tubes and mixed with 2 ml 0.6% TBA solution. The mixture was immersed in boiling water for 15 min and then quickly cooled to room temperature. The absorbance at 450, 532 and 600 nm was then measured with a UV-6100 UV–vis spectrophotometer (Metash Co., Ltd).

The content of soluble protein was determined by the Coomassie brilliant blue method [24]. Leaves (0.1 g) were ground into a homogenate with deionized water (5 ml), which was subsequently centrifuged (TDL-40B, Anting Scientific Instrument Factory, Shanghai) for 10 min at 3000 rpm. One millilitre of the supernatant was transferred to a test tube and diluted 5 times with deionized water (4 ml). One millilitre of the diluted solution was mixed with 5 ml of Coomassie brilliant blue G-250 (Shanghai Huishi Biochemical Reagent Co., Ltd). The absorbance was measured at 595 nm after 2 min with a UV-6100 UV–vis spectrophotometer (Metash Co. Ltd), and the protein content was determined via a standard curve [25].

The proline content was determined by the acid ninhydrin colourimetric method [37]. Afterward, 0.5 g of the sample was ground in 5 ml of 3% sulfosalicylic acid solution into a homogenate. After extraction in a boiling water bath for 10 min and cooling to room temperature, the mixture was centrifuged (TDL-40B, Anting Scientific Instrument Factory, Shanghai) at 4000 rpm for 10 min. Two millilitres of the supernatant was removed and added to 2 ml of ice-cold acetic acid and 3 ml of colour reagent (2.5% acidic ninhydrin). The mixture was subsequently immersed in a boiling water for 40 min. Afterward, 5 ml of toluene was added for extraction, and the absorbance was measured at a wavelength of 520 nm.

The chlorophyll content in the leaves of plant seedlings was directly measured with a SPAD-502 Plus chlorophyll meter (Konica Minolta Holdings, Inc.); a single leaf was measured three times [38].

### Data processing

Statistical analysis was performed with SPSS 16.0 statistical software (IBM SPSS Statistics, USA) using one-way analysis of variance (ANOVA), and multiple comparisons were performed on the basis of results of significance tests with the least significant difference (LSD) method. The significance level was 0.05, and all the experimental data were plotted with Origin 8.0 software (OriginLab Corp.). The experiments were repeated three times, and all of the results are presented as the means ± SEs.

## Supplementary information

**Additional file 1.**

**Additional file 2.**

**Additional file 3.**

**Additional file 4.**

**Additional file 5.**

## Data Availability

All data generated or analyzed during this study are included in this published article and its supplementary information files.

## Abbreviations

MDA: Malondialdehyde
TBA: Thiobarbituric acid
TCA: Trichloroacetic acid
